# Isolation and Characterization of Flavonoid Naringenin and Evaluation of Cytotoxic and Biological Efficacy of Water Lilly (*Nymphaea mexicana Zucc.*)

**DOI:** 10.3390/plants11243588

**Published:** 2022-12-19

**Authors:** Shajrath Din, Saima Hamid, Aadil Yaseen, Ali Mohd Yatoo, Shafat Ali, Kashif Shamim, Wael A. Mahdi, Sultan Alshehri, Muneeb U. Rehman, Wajaht A. Shah

**Affiliations:** 1 Department of Chemistry, University of Kashmir, Srinagar 190006, India; 2 Centre of Research for Development, University of Kashmir, Srinagar 190006, India; 3 National Centre for Natural Products Research, University of Mississippi, Oxford, MS 38677, USA; 4 Department of Pharmaceutics, College of Pharmacy, King Saud University, Riyadh 11451, Saudi Arabia; 5 Department of Clinical Pharmacy, College of Pharmacy, King Saud University, Riyadh 11451, Saudi Arabia

**Keywords:** pharmacology, *Nymphaea mexicana Zucc.*, secondary metabolites

## Abstract

Despite its limited exploration, *Nymphaea mexicana Zucc.* can be beneficial if pharmacology, isolation, and biological evaluation are given attention. It is an aquatic species that belongs to the family Nymphaeaceae. The thrust area of the work was the extraction, isolation, and biological evaluation of different extracts of the *N. mexicana Zucc.* plant. The primary goal of this research was to assess the antimicrobial, antioxidant, and anticancer activities of the extracts and to isolate the target naringenin compound. Comparative FT IR analysis of different extracts of this plant revealed the presence of functional groups of plant secondary metabolites, including polyphenols, flavonoids, terpenoids, esters, amines, glycosides, alkanes, alkaloids, fatty acids, and alcohols. Moderate free radical scavenging potential has been achieved for the various extracts via reducing power and DPPH assays. While cytotoxic activity was evaluated by colorimetric and lactate dehydrogenase cell viability tests on potent cancer cell lines. Lung adenocarcinoma epithelial cells (A-549), and breast cells (MC-7) were treated with MeOH extract. The antimicrobial activity against bacterial strains was evaluated using Gram-positive and -negative cultures, where maximum and minimum inhibition zones were recorded for different strains, including 1.6–25.6 μg/mL for *Streptococcus aureus*, using the agar well diffusion method. In addition, the anti-inflammatory activity of different extracts of *N. mexicana Zucc.* was evaluated in a nitrite radical scavenging assay with high concentrations of secondary metabolites, which are important against human pathogens and other diseases.

## 1. Introduction

Bioactive compounds derived from natural sources have a broader applicability in pharmacy and the food industry, and their applications, which now range from cosmetics and functionalized biomaterials to bioremediation and alternative fuels, have become more diverse as a result of the identification of unique properties in these substances [1]. It takes a lot of effort and time to create entirely new chemicals for medicinal use [2]. This has aided in the development of fields such as ethnopharmacology, which conducts systematic research and exploration of sources used historically as medicines [3], such as higher plants [4], microalgae, seaweeds [5], microorganisms [6], and fungi [7]. Overproduction of many reactive oxygen species, including oxygen radicals and non-free radical species, is thought to be the main cause of oxidative stress, which has been linked to a number of diseases such as cancer, tissue damage in rheumatoid arthritis, and atherosclerosis [8]. Edible plants are the main sources of antioxidants, which the body uses to protect itself from damage caused by free radical-induced oxidative stress, and plants are a typical source of medications [9]. The antitumor effects of phytochemicals range from genotoxic effect suppression to protease inhibition and cell growth inhibition, improved antioxidant activity, signal transduction pathways, and protection of intracellular infrastructures to control apoptosis [10].

Nymphaeaceae has been traditionally used for dyspepsia, piles, diarrhea, and urinary ailments. The rhizome of this plant has been used for cystitis, nephritis, enteritis, fevers, and insomnia [11]. In India, China, and Nepal, many species of this plant, *Nymphaea,* are known to act as functional drugs. *N. mexicana Zucc.* is an aquatic plant commonly known as the yellow water lily, which belongs to the family Nymphaeaceae, comprised of 5–6 genera and 60–95 species. Among them, approximately 70 species and 6 genera are spread worldwide [12]. The water lily, *Nymphaea*, is an annual or perennial herb, and this species has the potential to grow rapidly and become weedy in dams, ponds, and rivers. The yellow water lily is native to the southern United States and Mexico, but it has spread to many other countries through the ornamental plant trade and is considered a religious plant. The phytochemistry and biology of *Nymphaea* plants have been investigated for various uses, including treating diabetes and cancer, as well as for antioxidant, and hepatoprotective activities [13,14,15]. The reports from the literature indicated that the *Nymphaea* plant contains multiple active compounds, including flavonols, flavonoid glycosides, triterpenes, anthocyanins, acylated anthocyanins, alkaloids, hydrolysable tannins, and saponins. The extracts of the rhizomes and seeds, have immunomodulatory activity. The extracts of the stalks have an antipyretic effect. Extracts of the flowers, stamens, and leaves have shown antioxidant effects [16,17]. In traditional Sudanese medicine, the leaves of the water lily have been used as a remedy for dysentery, as an antibacterial, and to treat tumors and other disorders [18]. The different species of *Nymphaea* in Africa are being used in the management of cancer. Nymphaea species are reported to possess anti-inflammatory and anti-diabetic properties [19], and plants of Nymphaea species are recognized to be larvicidal, sedative, anti-microbial, and anti-hyperlipidemic [20].

Therefore, it was essential to conduct the current study to learn more about the valuable bioactivity of extracts from *N. mexicana Zucc.* aerial parts so that this plant may be used in large quantities. Based on the data, it was discovered that the hexane extract had a considerable amount of antibacterial activity. In contrast, the dichloromethane and methanol extracts had substantial amounts of antioxidant, cytotoxic, and anti-inflammatory properties.

## 2. Results

### 2.1. Isolated Compound Naringenin

Naringenin [(2S)-5,7-Dihydroxy-2-(4-hydroxyphenyl)-2,3-dihydro-4H-chromen-4-on] is the obtained compound having molecular formula C_15_H_12_O_5_ with melting point 253–254 °C. IR spectrum showed the presence of -OH 3290, 3117 cm^−1^, ketone at (C=O) 1626 cm^−1^. Solvent separation system: 40% MeOH: DCM. ^1^H-NMR measurements were performed by a Bruker Avance spectrometer at operating frequency of 500 MHz using Dimethyl sulphoxide (DMSO) as solvent.

The ^1^H-NMR spectrum comprising of two singlets of H atoms, in ring A of the two flavanones, corresponding to hydrogens H_6_ and H_8_. The ^13^C NMR-DEPT spectrum of the compound presents 15 carbon signals including seven quaternaries; seven are methine group and one methylene carbon atoms. The characterizing of flavanone skeleton is presented between the two carbon molecules of an oxymethynic group with chemical shifts at 𝛿 79.0 and 42.61. The isolated molecule was identified as naringenin, a flavonoid, based on spectrum evidence and comparison of physical data with published values [21,22,23]. The comparative shift values are given in Table 1 with supportive Supplementary Figure S1).

### 2.2. FTIR Spectral Analysis

**Figure 1:**
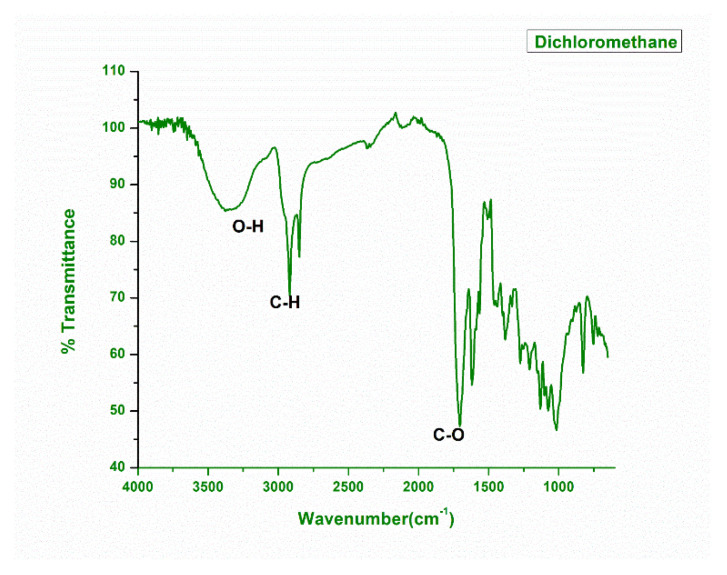
FTIR analysis of DCM extract of plant *Nymphaea Mexicana Zucc*.

The DCM, MeOH, and n-Hexane leaf extract *N. mexicana Zucc.* was subjected to FTIR analysis in order to confirm the presence of major functional groups in these extracts. The obtained spectral corresponding data were plotted between % transmittance and wavenumber (cm^−1^) using the software OriginPro 8.5. It was found that in dichloromethane extract, the major absorption peaks 3000 cm^−1^ (corresponding to C-H stretching), 1800 cm^−1^ (corresponding to C-O stretching) and 3200 cm^−1^ (corresponding to O-H stretching) (Figure 1).

The presence of an O-H peak reflects the presence of polyphenols in this extract, in which the O-H group interacts with the DCM solvent through hydrogen bonding. Similarly, the importance of the C=O peak suggests the presence of those compounds that contain oxo functional groups in their structures, such as aldehydes and ketones.

**Figure 2:**
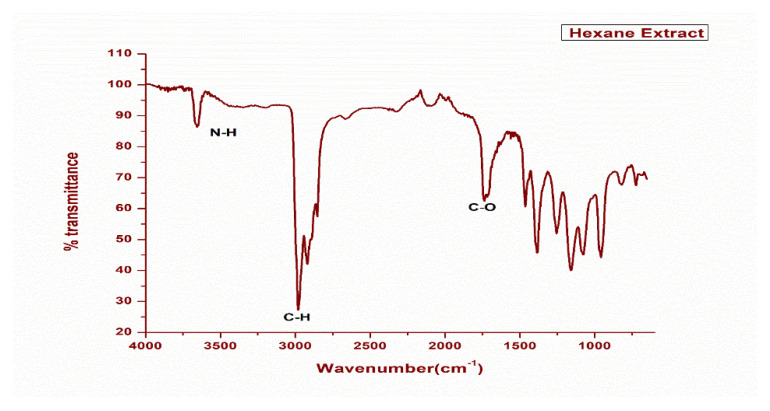
FTIR spectral analysis of hexane extract of plant *Nymphaea Mexicana Zucc*.

**Figure 3:**
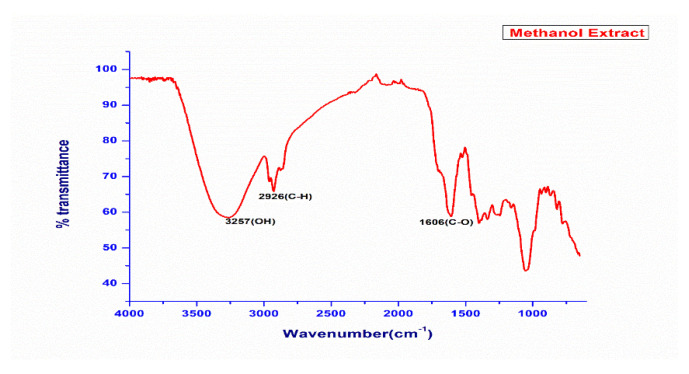
FTIR spectral analysis of methanol extract of plant *Nymphaea Mexicana Zucc*.

Similar FTIR analysis was performed on crude hexane extract of the plant material, and it was found that the prominent absorption peaks were 3600 cm^−1^ (corresponding to N-H stretching), 3000 cm^−1^ (corresponding to C-H stretching in alkene), 2914 cm^−1^, 2847 cm^−1^ (corresponding to C-H stretching in alkane). The other prominent peaks in the low frequency region in this extract are 1700 cm^−1^ (C=O stretching). The fingerprint region of this extract consists of peaks 1584 cm^−1^, 1401 cm^−1^, 1274 cm^−1^, 1215 cm^−1^, 1177 cm^−1^, 1148 cm^−1^, 1021 cm^−1^. All these peaks are indicating the presence of flavonoids, amines, carboxylic acids, ketones, alkenes, and terpenoids, which are non-polar in nature. The prominent absorption peaks in the MeOH extract were 3257 cm^−1^ (corresponding to O-H stretching), 2926 cm^−1^ (corresponding to C-H stretching in alkene), and 1606 cm^−1^ (corresponding to C-O) stretching. The FTIR absorption spectrums for comparison of both methanol and hexane extracts of this plant is summed up in Supplementary Table S2a,b along with respective spectra as shown in Figure 2 and Figure 3.

### 2.3. Biological Efficacy

#### 2.3.1. Anti-Microbial Activity

The preliminary antibacterial activity of different extracts of the plant was performed against five bacterial strains (Table 2).

#### 2.3.2. Cytotoxic Evaluation

DCM and MeOH extracts were tested against lung and breast cancer cell lines by following the methods of lactate dehydrogenase and tetrazolium reduction assay, and the observations are given in Table 3 and Table 4.

DPPH and FRAP assays were carried out to determine the free radical scavenging potential of extracts from the experimental plant in order to obtain an exact figure for the neutralization of radical generation due to oxidative stress.

### 2.4. 2,2- Diphenyl-1-picryl-hydrazyl Evaluation

The substances in the extract that might exhibit antioxidant effects will oxidize themselves and change DPPH into DPPH_2_. Due to the decline in absorbance measured at 517 nm, the amount to which this reaction occurs is readily visible. The antioxidant assay was used to assess the radical scavenging properties of a *N. mexicana Zucc.* extract using the DPPH free radical assay (Table 5).

#### FRAP Assay

The capacity of extract and pure chemicals to change ferric ion (Fe^3+^) to ferrous ion (Fe^2+^) by a single electron transfer redox reaction was used to assess the FRAP. The antioxidant activity was assessed using the following concentration range: 10, 20, 30, 50, 70, and 100 g/mL. Results were contrasted with those of ascorbic acid, the control, which demonstrated a maximal inhibition of 80.7% at a concentration of 100 g/mL. The absorbance increases with concentration, which suggests that concentration increases reducing power. This approach also revealed that methanol extract and DCM extract had higher levels of activity, with activity values of 68% and 48%, respectively, at a concentration of 100 g/mL, as shown in Table 6.

### 2.5. Isolated Compound

Olsen and co-workers isolated narengenin from *Mentha aquatica* L. using 70 percent ethanolic extract where highest inhibitory activity have been obtained and known to be potent MAO-inhibitor, while characterization was confirmed by ^1^H, ^13^C and ^13^C-DEPT NMR and optical rotation. Using the heat reflux technique, a high yield of naringenin 23-SHR (35.80 1.79 g/g) was generated from the segmental portion of grapefruit (*Citrus paradisi* L.) [24,25].

### 2.6. Biological Evaluation

#### 2.6.1. Anti-Microbial Activity

Except for *S. typhii*, all of the extracts were shown to exhibit zones of inhibition against the tested strains. However, the largest inhibition zones were observed to be present against *Staphylococcus aureus* (22 mm) and *K. pneumonia* (24 mm). The extracts were proven to be effective against the tested strains; as a result, MIC was established. All of the examined microorganisms have MIC values between 1.6 and 25.6 mg/mL, while *E. coli* and *S. aureus* had the lowest values. Therefore, against the studied strains, plant extracts had a wide range of antibacterial effects. Akinjogunla and co-workers [26] carried out an in vitro anti-microbial activity of EAOtc extracts of *N. lotus* where highest susceptibility was found against *Streptococcus sp.* followed by *S. aureus* and *E. coli* using streptomycin as standard drug.

#### 2.6.2. Cytotoxic Assay

**Figure 4:**
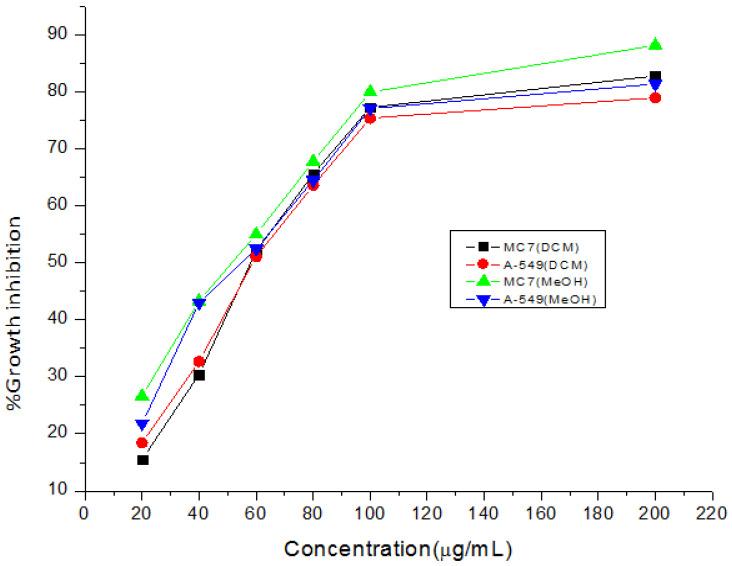
Anticancer activity of extracts of *N.mexicana Zucc.* by MTT assay.

**Figure 5:**
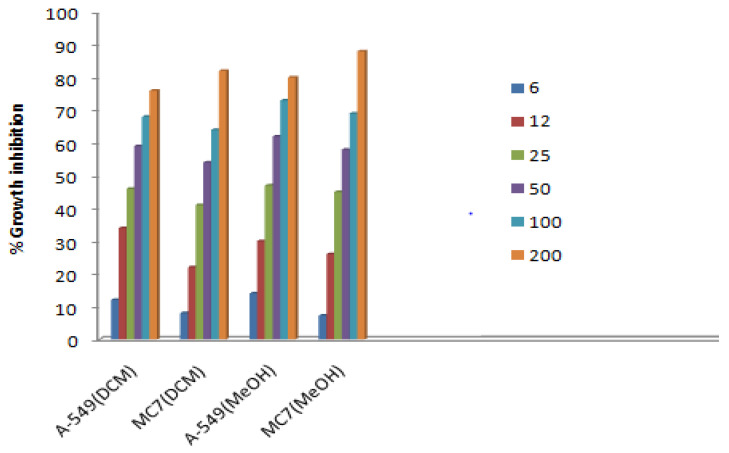
In vitro cancer activity of dicholoromethane and methanol extract of *N. mexicana Zucc.* against MC7 and A-549.

Every sample that was examined was confirmed to be functional. However, the methanol extract’s greatest activity was discovered in the MTT experiment against lung cancer cell line and breast cancer cell line with (IC_50_) value of 42.8 μg/mL, and 43.3 μg/mL respectively, as is evident from the graph (Figure 4). LDH assay was performed on the above-mentioned cell lines, among which the methanol extract was found to be highly active against A-549 cell line which exhibited growth inhibition of 73% at a concentration of 100 μg/mL, with IC_50_ values 29 μg/mL shown in the graph. It is clear from the results that the methanol extract showed an enhanced anticancer effect with the increase in concentration of extract. To the best of our knowledge, this extract’s anticancer efficacy has never been documented. It would be crucial for pharmaceutics to use this extract instead of manufactured ones because of its strong cytotoxic potential, which might have negative side effects (Figure 4 and Figure 5). When it came to preventing lipid peroxidation, the extract was six times more effective than gallic acid. Similar to diclofenac, the extract reduced the extent of oedema in rat paws in a dose-dependent manner. It also significantly reduced ESR and wheel diameters. Both the Jurkat cell line and the MCF-7 cell line were cytotoxic to the extract (IC_50_ = 87.29 g/mL and 155.00 g/mL, respectively), with a higher selectivity for the Jurkat cell line. It’s intriguing that the extract has a modest cytotoxicity against the common Chang liver cell line (IC_50_ = 204.20 g/mL) [27].

#### 2.6.3. Antioxidant Activity

##### DPPH Assay

**Figure 6:**
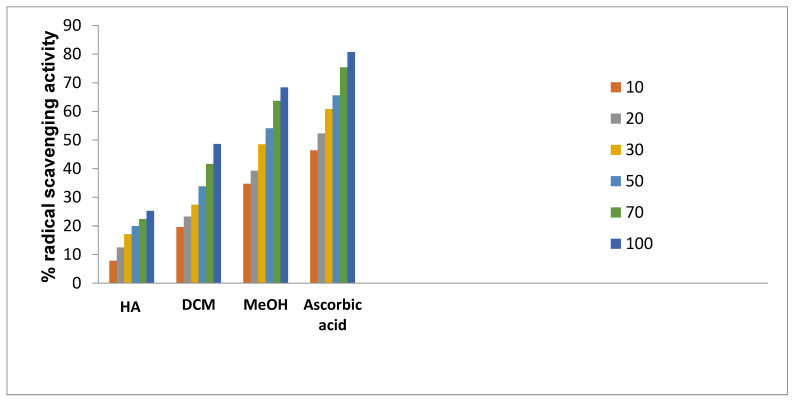
DPPH free radical scavenging activity of different extracts. [HA: hexane, DCM: dichloromethane, MeOH: methanol extracts of *N. mexicana Zucc.* and ascorbic acid is a positive control].

The result was observed at different concentrations 10–100 μg/mL. The methanol extract of *N. mexicana Zucc.* was found to be more active than pet-ether extract. 72.77% and 62% RSA of extract were due to active molecules which were responsible to oxidize DPPH to DPPH_2_, thus representing presence of antioxidant rich potential as also shown in Figure 6. N’guessan and co-workers [27] identified the phenolic, flavonoid, and elemental components of *N. lotus* leaves’ hydro-ethanolic extract (NLE), as well as the cytotoxic, anti-inflammatory and anti-oxidant capabilities of the NLE. The extract was two times more effective than butylated hydroxytoluene at scavenging DPPH radicals and contained significant amounts of phenolic and flavonoid components (BHT). NLE was, however, three and six times less effective than ascorbic acid and BHT, respectively, at converting Fe^3+^ to Fe^2+^. The plant extract has a six-fold greater inhibitory effect on lipid peroxidation than gallic acid. The *N. nouchali* hydroethanolic seed extract and tocopherol at 1.95 g/mL inhibit the DPPH radical scavenging potential at 8.56% and 16.82% respectively, additionally IC_50_ values were 42.82 g/mL and 27.82 g/mL respectively. It was discovered that the hydroethanolic seed extract from *N. nouchali* has less scavenging power than vitamin E. At 1000 g/mL of *N. nouchali* hydrorthanolic SE, the % inhibition was found maximum at 89.47 g/mL [28,29].

##### Reducing Power Assay

**Figure 7:**
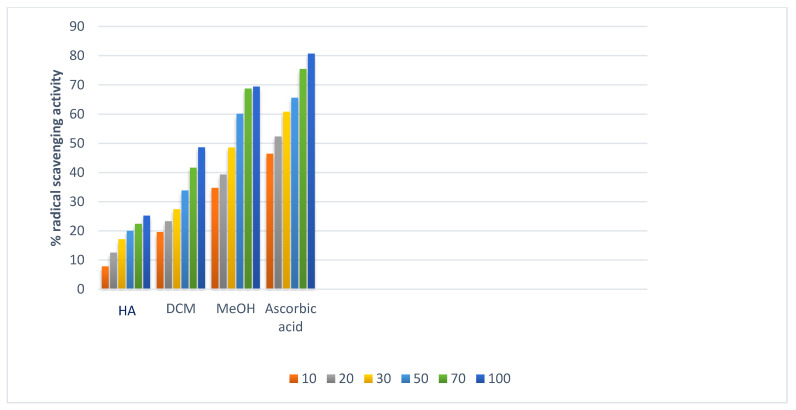
Reducing power of extracts of *N. mexicana Zucc*.

From our observed results it has been found that with increasing concentration the free radical neutralizing capacity increases with increasing absorbance. 100 µg/mL concentrations for MeOH and DCM showed potent results at 68 percent and 48 percent respectively (Figure 7).

## 3. Discussions

A mounting bulk of evidence suggests that oxidative stress and inflammation are two primary mechanisms that contribute to cancer initiation and progression. We intended to contribute to pharmacological aspects by looking into the antioxidant, anti-inflammatory, and cytotoxic activities of *N. lotus* (a medicinal plant traditionally used to treat cancer patients) in relation to its phytochemical and elemental constituents. Phytochemical analyses revealed that flavonoids and phenolic compounds are the most abundant bioactive metabolites in Nymphaea species, with *N. lotus* containing very special macrocyclic flavonoids [30,31].

Important natural substances called flavonoids have a range of biological effects. An essential group of flavonoids is the citrus flavonoid family. This flavonoid group, including naringin and naringenin, was discovered to have potent anti-inflammatory and antioxidant properties. Numerous lines of research point to the benefits of naringin supplementation in managing metabolic syndrome, diabetes, hypertension, and obesity [32,33,34].

Numerous molecular pathways underlying its advantageous properties have been discovered [35]. At first, grapefruit leaves and celery seeds were found to contain naringenin in isolation. The process of manufacturing ketchup changes the substance found in tomato skin, naringenin-chalcone, into the more toxic substance, naringenin [36,37]. The inactive naringenin, called “naringin,” is initially present in the body [38,39,40,41]. Naringenin is distributed differently in the fruit’s various portions depending on the levels of biosynthetic enzymes. For instance, higher levels of flavanone 3-hydroxylase gene expression and chalcone synthesis than the chalcone isomer are found in the peel of *Solanum lycopersicum*, which suggests that naringenin has accumulated there in large amounts [42]. Researchers used rats to determine whether naringenin inhibits glucosidase and the potential applications of this inhibitory action [43]. With IC_50_ values of 0.0065 and 0.384 mM, respectively, naringenin was discovered to inhibit both yeast and mammalian glucosidase. Naringenin competitively inhibited mammalian glucosidase, indicating that it binds to the enzyme’s active site. Naringenin had more noticeable inhibitory effects on postprandial hyperglycemia in diabetic rats after maltose and sucrose challenges [44].

In DPPH assays, the methanolic extract of *N. mexicana Zucc.* demonstrated 72.4% radical scavenging potential at 100 µg/mL compared to ascorbic acid (80%), while the obtained reducing power activity was 68 per cent. Antioxidant substances in a reducing power assay cause the iron in ferric chloride to change from its oxidative state (Fe3+) to ferrous (Fe^2+^). Ascorbic acid or BHT was more effective than the extract at converting Fe^3+^ to Fe^2+.^ A related investigation discovered that the *Nyctantes arbour* hydro-alcohol and chloroform extracts had reducing power properties comparable to ascorbic acid [45].

India is ranked third in the world with an increasing rate of cancer, mainly breast, lung, and cervix carcinoma. As a result, the need of the hour is to include foods that cure these types of diseases by identifying agents that will aid in the fight against such cancers. MCF-7, a human adenocarcinoma breast cancer cell line, has glucocorticoid, progesterone, and oestrogen receptors. In our study, the methanolic extract showed 73% inhibition against the A-549 lung cancer cell line at a concentration of 100 μg/mL through the LDH assay, while IC_50_ values of 42.8 μg/mL and 43.3 μg/mL were achieved for the lung and the breast cancer cell lines, respectively. These two cell lines are good models for testing cytotoxic (anti-cancer) drugs. Hence, synthetic drugs can replace naturally existing food sources such as *N. mexicana Zucc.* However, similar results have been observed for antimicrobials. These findings support previous findings for their potential cytotoxic properties, such as *N. alba*, *N. mexicana* and *N. lotus* [46]. These plants may have cytotoxic properties due to their phytochemical and elemental compositions, which may explain why *N. mexicana Zucc*. has historically been used to treat cancer. The strong O-H stretching in both the IR spectrums were found in the range of 3000 to 3800 cm^−1^ due to presence of functional groups including carboxylic acids, ketones, amines, polyphenols, terpenoids, tertiary and secondary alcohols. However, our findings are in line with Abubaker and co-workers, [47] who carried out the FTIR analysis of petroleum ether oil extract for the plant *Ziziphus spina-christi* hence, revealed the presence of alkanes, alkenes, carboxylic acids, ketones, amines, alcohols, and phenols as major ones. While examining the methanol extract of *Ceropegia juncea*, Visveshwari et al. [48] discovered the presence of alcohols, aldehydes, alkynes, alkenes, esters, and amines groups. This justification suggests that *N. mexicana Zucc.* has a variety of therapeutic phyto-constituents that have a variety of bioactivities, including antibacterial, antifungal, antioxidant, anti-inflammatory, anti-diabetic, anti-aging, anticancer, hepatoprotective, hypercholesterolemic, antihistaminic, anticoagulant, diuretic, etc. The identification of several phyto-constituents in the n-hexane, methanol and DCM plant extracts of *N. mexicana Zucc.,* hence demonstrates significant therapeutic implications.

## 4. Materials and Methods

### 4.1. Collection, Preparation of Plant Materials and Crude Extract

#### 4.1.1. Plant Material

*N. mexicana Zucc.* plant was collected from Dal Lake, Srinagar. The plant specimen was identified by Curator, CBT, University of Kashmir vide voucher no. 3734–(KASH) Herbarium.

#### 4.1.2. Preparation of Extracts

The collected fresh plant samples were rinsed with water, shade dried, and powdered in an electric grinder. Dried shoot powder (100 g) was successfully obtained after following hot extraction in soxhlet extractor using solvents of escalating polarity from hexane, dichloromethane and methanol for each respectively. Plant material was repeatedly extracted until a colorless solvent was attained. The crude extracts were obtained using a Rota evaporator (RV-8-S096), which was fully dehydrated, weighed, and kept at 4 °C in sealed, sterile vials for further use. The methanolic extract was used for both column chromatographic and FTIR analysis, while hexane and DCM extracts were employed for the FTIR and in vitro biological evaluations for comparative efficacy.

#### 4.1.3. Fourier Transform Infrared Spectroscopic Analysis

FTIR spectrometer (Agilent Technologies, Santa Clara, CA, USA, carry 630 FTIR) was used to obtain FTIR spectra. This instrument was set up as a Windows-based device and connected to the OPUS operating system software (OriginPro 8.5, Northampton, MA, USA). The graph was plotted between **%** transmittance and wave number (cm^−1^) in order to confirm the presence of major absorption peaks.

### 4.2. Column Chromatography for Compound Separation

Nearly forty grams of MeOH extract were subjected to column chromatography which was packed with solid powder of silica gel (60–120 mesh size), later followed by solvent run enduring increasing polarity order from 1 percent, 2 percent and so on for hexane, ethyl acetate, and methanol in order to isolate compounds. The solvent percentage followed for column were from 1% hexane to 100%; 1% EtOAc to 100% according to increasing following trend of polarity. However, the target compound was isolated at solvent system of 30% EtOAc; hexane. 

### 4.3. Biological Evaluation of Extract of N. mexicana Zucc

#### 4.3.1. Antibacterial Activity

Institute of Microbial Technology (IMTECH), Chandigarh, India, procured the different bacterial strains (*Escherichia coli* MTCC 641, *Pseudomonas aeruginosa* MTCC 2868, *Staphylococcus aureus* MTCC 96, *Klebsiella pneumoniae* MTCC, and *Salmonella typhii* MTCC 341) in order to go for preliminary antibacterial screening of *N. mexicana Zucc.* For the development of bacterial strains, liquid nutritional agar (LNA) was employed, which was retained for incubation at 37 °C for 24 h, stored at 4 °C in the refrigerator, and utilized as stock cultures.

Testing for bacterial susceptibility to various antibiotics was performed using the agar well diffusion assay with some modifications. Bacteria were inoculated into 25 mL of Muller–Hinton Agar. The tubes containing agar were shaken and transferred into Petri plates. After waiting for 10 min for the setting of the agar, the wells were punched on the plates. A capillary micropipette was used to fill these wells with 25 μL of extract. The bacteria were incubated at 25 °C for 18 h. The antibacterial activity was determined by measuring the width of the zones of inhibition against the indicator strains or test microorganisms with a vernier scale. Streptomycin sulphate (1000 mg/L) is the positive control that is employed [49].

##### Evaluation of Minimum Inhibitory Concentration

The MIC was assessed using the agar dilution method with some modifications. Agar medium was incorporated into tween-20 (0.5%) for the proper dissolution of extracts in the agar. To ensure microbe-free conditions, 20 mL of agar in a boiling tube was sterilized in an autoclave. DMSO solvent was utilized for extract dilution in order to acquire a series of concentrations ranging from 0.2–25.6 μg/mL. The temperature of the boiling tubes was reduced to 38 °C, and extract concentrations ranging from 0.2–25.6 μg/mL were added to agar. Each bacterium containing 3 μL inoculum was added into the petri-plates, prepared with concentration of 0.5 × 105 CFU/mL and its turbidity was adjusted in the range of 0.08–0.13 at 625 nm. For determining the MIC, the inoculated petri plates were incubated at 37 °C for 18 h. All of the experiments were repeated three times, and the inhibition of bacterial growth in the agar plates containing the various extracts was measured by comparing it to bacterial growth on a blank plate (containing only agar and bacteria). The MIC on the agar plate was determined by the last concentration of the extracts that suppresses visible bacterial growth [50].

#### 4.3.2. Anticancer Activity

##### Cell Lines and Culture

The anti-proliferative impact of the extracts was tested using the MTT (3-(4,5-dimethylthiazol-2-yl)-2,5-diphenyltetrazolium bromide, a tetrazole) technique. To prevent confluence of the culture during treatment, an adequate amount of rampant colonial cells was utilized. The cell lines A-549 and MC-7 were cultivated in 96-well plates under standard culture conditions, with the necessary cell quantity in 100 µL medium per well and were left to adhere for 12 h. Different cell types were exposed to extracts at concentrations ranging from 20 to 250 g/mL in an effort to determine the lowest dose at which the introduced extracts inhibit cell growth in various cell lines. Streptomycin was utilized as positive control in the cytotoxic assay at a concentration of 1 to 5 g/mL, and dimethyl sulfoxide solvent was used to dilute the extracts and served as the experimental control. The MTT test was applied to each tier and was kept at optimum temperature for three hours to measure the cell growth after 48 h of treatment. To dissolve the formazan crystals, 150 µL of MTT solvent (4 mM Hcl, 0.1% Nonidet P-40, both in isopropanol) was applied to each well after the medium had been aspirated. Three duplicates of each sample were taken. At a wavelength of 570 nm, the absorbance of plates was assessed using an ELISA reader [51,52,53].

##### LDH Assay

A 24-well plate was used for culturing of cells. Each well was filled with a medium of 500 μL. Cells cultured for 24 h at a seeding density of 2 × 10^5^ were treated with plant extracts. The LDH activity was analyzed by taking out the medium of 200 μL. LDH assay kit was used for determining total LDH activity. Sonication was used for cell disruption. The LDH release (%) was calculated by the formula:

LDH release = [Absorbance of supernatant/Absorbance of supernatant and cell lysate] × 100

#### 4.3.3. Antioxidant Activity

##### DPPH Assay

To 1 mL (0.5 mM DPPH), different concentrations (15–250 g/mL) were added, and the final amount was increased to 3 mL by adding methanol. The aforementioned combination was well mixed, covered, and kept at room temperature in the dark for 30 min. A spectrophotometer (NW-6850) was used to test the mixture’s absorbance at 517 nm. Three copies of each experiment were run. When the absorbance drops, there are fewer DPPH radicals present, which results in an increase in DPPH-RSA [43,44,45,46,47,48,49].

The following equation was used to determine the radical scavenging effect:

DPPH radical scavenging effect (%) = ([A control − sample]/A control] × 100)

When L-ascorbic acid is used as a positive control in the extract, sample B’s absorbance is used as a control instead of sample A’s absorbance of the blank sample. Plotting was performed between the extract concentration and the percentage of scavenging activity.

##### FRAP

The potassium ferricyanide [K_3_Fe(CN)_6_] and sodium phosphate buffer was combined with the 1% *w*/*v* plant extract in 2.5 mL (10–100 g/mL) (pH 6.6). The resulting liquid was vortexed and incubated for 20 min at 50 °C after 2.5 mL of tricarboxylic acid (10% *w*/*v*) was added. The mixture was centrifuged for 10 min at 3000 rpm to remove the top layer, and then 2.5 mL of distilled water and 0.5 mL of FeCl_3_ (0.1% *w*/*v*) were added. Using a spectrophotometer (JW-6850), the absorbance was determined at 700 nm in comparison to a blank sample using ascorbic acid as the positive control [21]. All the reagents were present in the blank solution, which was extract-free. A greater reducing power may be calculated by looking at the absorbance value. To obtain the reduction power, the following equation was used:

% reducing power = (1 − [1-absorbance of control/absorbance of sample]) × 100

## 5. Conclusions

*Nymphaea mexicana Zucc.* is an aquatic plant that has mud-anchored perennial rhizomes or rootstocks. It is commonly available throughout Europe, Asia, North Africa, and Russia. *N. mexicana Zucc.* exhibited significant biological activities, including antioxidant, antimicrobial, anticancer, and anti-inflammatory properties. Our results indicate that this plant showed promising anticancer activity against the cell lines A-549 and MC-7 by MTT assay and LDH assay, with IC_50_ values of 46 and 29, respectively. Moreover, the methanol extract of this plant exhibits moderate anti-inflammatory potential. Hence, this plant can be used in the formulation of anti-inflammatory and cytotoxic agents that can treat various diseases. Therefore, this study may be of immense use to the researchers to provide new opportunities in the future in the field of chemical, taxonomic, and bioactive approaches. As a result, it will be beneficial to discover some novel antibiotics against various infectious diseases and provide appealing alternative sources to consider. All the extracts have shown good biological activity and can be positively exploited for treatment strategies against different diseases. From the above studies, it has been proven that this plant is an excellent antioxidant and has anti-inflammatory and anticancer properties.

## Figures and Tables

**Table 1:** ^13^C and ^1^H NMR chemical shift values (ppm) of naringenin.

Position	^13^C	^1^H
2	79.05	5.30
3a	42.61	3.09
3b	42.61	2.67
4	196.38	-
5	164.03	-
6	95.68	5.90
7	166.90	-
8	94.80	5.90
9	163.45	-
10	101.94	-
1′	129.68	-
2′	127.68	7.31
3′	114.93	6.83
4′	157.57	-
5′	114.93	6.83
6′	127.68	7.31

**Table 2:** Antibacterial activity of various extracts of *N. mexicana Zucc*.

Microorganisms	Zone of HA Extract ^a^	Inhibition DCM Extract ^b^	(mm) MeOH Extract ^c^	Antibiotic	MIC
*Escherichia coli* MTCC 443	19 ± 0.5	18 ± 0.45	17 ± 0.6	25 ± 0.45	>3.2
*Klebsiella pneumonia* MTCC 19	24 ± 0.1	19 ± 0.6	20 ± 0.27	28 ± 0.4	>1.6
*Pseudomonas aeroginosa* MTCC1688	19 ± 0.38	18 ± 0.45	20 ± 0.14	25 ± 0.42	>3.2
*Salmonella typhii* MTCC 341	Not active	18 ± 0.6	10 ± 0.8	25 ± 0.6	>25.6
*Staphylococcus aureus* MTCC 96	22 ± 0.28	23 ± 0.18	20 ± 0.8	25 ± 0.8	>1.6

^a^ HA: Hexane extract, ^b^ DCM: dichloromethane and ^c^ MeOH: methanol extracts zone of inhibition in (mm) of *N. mexicana Zucc*.

**Table 3:** In vitro anticancer activity by MTT assay of *N. mexicana Zucc.* extract.

DCM Extract Concentration (μg/mL)	% Growth Inhibition of Lung Cancer Cell Line (A549)	IC_50_ (μg/mL)	% Growth Inhibition Breast Cancer Cell Line (MC-7)	IC_50_ (μg/mL)
20	18.4		15.5	
40	32.7		30.4	
60	51.1	59	52.1	60
80	63.6		65.6	
100	75.4		77.3	
200	79.0		82.8	
				
**Methanolic extract concentration (μg/mL)**	**% growth inhibition of lung cancer cell line (A549)**	**IC_50_ (μg/mL)**	**% growth inhibition breast cancer cell line (MC-7)**	**IC_50_ (μg/mL)**
20	21.8		26.6	
40	43.0		43.3	
60	52.5	46	55.0	48
80	64.5		67.8	
100	77.1		80.8	
200	81.4		88.2	

**Table 4:** In vitro anticancer activity by LDH assay of *N. mexicana Zucc.* extract.

	Tissue Type Model Type	Lung A-549	Breast MC7
DCM Extract Concentration (μg/mL)	6	12	8
	12	34	22
	25	46	41
	50	59	54
	100	68	64
	200	76	82
		
MeOH Extract Concentration (μg/mL)	6	14	7.3
	12	30	26
	25	47	45
	50	62	58
	100	73	69
	200	80	88

Free radical scavenging assay.

**Table 5:** %RSA via DPPH of *N. mexicana Zucc.* extracts.

Concentration (μg/mL)	HA ^a^	DCM ^b^	MeOH ^c^	Ascorbic Acid ^d^
10	3.64	8.54	15.90	46.4
20	8.21	24.01	36.90	52.3
30	12.01	28.53	46.56	60.8
50	13	47.71	58.12	65.6
70	17.40	54.33	69.07	75.4
100	28.35	61.74	72.77	80.7

RSA = radical scavenging activity; ^a^ percentage of hexane extract of *N. mexicana Zucc.,* ^b^ percentage of *N. mexicana Zucc.*, ^c^ percentage of methanol extract of *N. mexicana Zucc.,* ^d^ percentage of positive control ascorbic acid.

**Table 6:** Ferric reducing activity of three extracts of *N. mexicana Zucc*.

Concentration (μg/mL)	HA ^a^	DCM ^b^	MeOH ^c^	Ascorbic Acid ^d^
10	7.82	19.6	34.7	46.4
20	12.5	23.3	39.3	52.3
30	17.1	27.4	48.5	60.8
50	20.0	33.8	54.1	65.6
70	22.4	41.6	63.7	75.4
100	25.2	48.60	68.4	80.7

Ferric reducing activity: Concentration of hexane, dichloromethane and methanol in μg/mL, ^a^ percentage of hexane extract of *N. mexicana Zucc.,* ^b^ percentage of *N. mexicana Zucc.*, ^c^ percentage of methanol extract of *N. mexicana Zucc.,* ^d^ percentage of positive control ascorbic acid.

## Data Availability

Not applicable.

## Supplementary Materials

The following supporting information can be downloaded at: https://www.mdpi.com/article/10.3390/plants11243588/s1, Figure S1: NMR spectra of Naringenin. The hydrogen of the hydroxyl of carbons C-5 and C-7 in the naringenin molecule is responsible for displacements at 7.21 and 6.83 ppm, respectively. ^1^H-NMR measurements were performed by Bruker Avance spectrometer at operating frequency of 500 MHz using Dimethyl sulphoxide (DMSO) as solvent; Table S1: Assigned peaks and corresponding functional moieties via FTIR analysis present in hexane extracts of plant *N. Mexicana Zucc.;* Table S2. FT IR analysis, assigned peaks and corresponding functional moieties present in methanol extract of plant *N.mexicana Zucc.*
