# Supplementary material for: Isolation and Characterization of Flavonoid Naringenin and Evaluation of Cytotoxic and Biological Efficacy of Water Lilly (Nymphaea mexicana Zucc.)
Source: Plants (Basel). 2022 Dec 19;11(24):3588. doi: 10.3390/plants11243588 (PMC9780907; doi:10.3390/plants11243588)
Supplement: Supplementary file 1 [file plants-11-03588-s001.zip › plants-2021107-supplementary.pdf]

plants

MDPI

Supplementary Materials

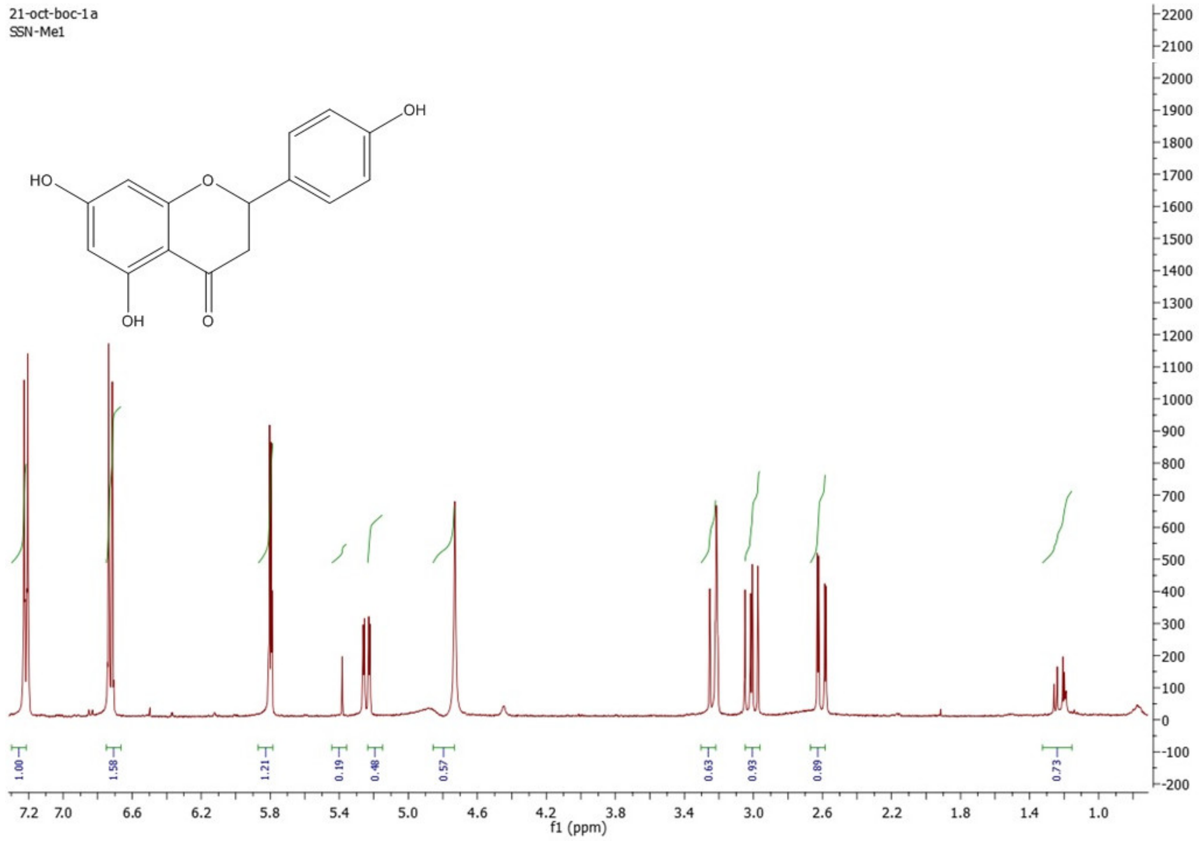

Figure S1. NMR spectra of Naringenin. The hydrogen of the hydroxyl of carbons C-5 and C-7 in the naringenin molecule is responsible for displacements at 7.21 and 6.83 ppm, respectively.^{1}H-NMR measurements were performed by Bruker Avance spectrometer at operating frequency of 500 MHz using Dimethyl sulphoxide (DMSO) as solvent.

Table S1. Assigned peaks and corresponding functional moieties via FTIR analysis present in hexane extracts of plant *N. Mexicana Zucc.*

S.No	Absorption frequency(cm^{-1})	Appearance	Functional Group	Compound class
1	3746	medium, sharp	O-H stretching	alcohol
2	3671	strong, broad	O-H stretching	alcohol
3	3377	strong	O-H stretching	Alcohol(intermolecular bonded)
4	2918	medium	C-H stretching	alkane
6	2366	Strong, broad	N=C=O stretching	isocyanate
7	2117	weak	C≡C	Alkyne (mono-substituted)
8	1703	strong	C=O stretching	Conjugated acid(dimer)
9	1617	medium	C=C stretching	Conjugated alkene
10	1334	medium	O-H bending	alcohol
11	1505	strong	N-O stretching	Nitro compound
12	1438	medium	O-H bending	Carboxylic acid
13	1379	Strong	S=O stretching	Sulfonic acid
14	1274	strong	C=O stretching	alkyl aryl ether
15	1207	strong	C=O stretching	ester
16	1129	strong	C=O stretching	aliphatic ester
17	1037	strong	S=O stretching	sulfoxide
18	1013	Strong, broad	CO-O-CO stretching	anhydride
19	823	medium	C=C stretching	Alkene(tri-substituted)

Table S2. FT IR analysis, assigned peaks and corresponding functional moieties present in methanol extract of plant *N.mexicana Zucc.*

S.No	Absorption frequency(cm^{-1})	Appearance	Functional Group	Compound class
1	3746	medium, sharp	O-H stretching	alcohol
2	3671	strong, broad	O-H stretching	alcohol
3	3377	strong	O-H stretching	Alcohol(intermolecular bonded)
4	2918	medium	C-H stretching	alkane
6	2366	Strong, broad	N=C=O stretching	isocyanate
7	2117	weak	C≡C	Alkyne (mono-substituted)
8	1703	strong	C=O stretching	Conjugated acid(dimer)
9	1617	medium	C=C stretching	Conjugated alkene
10	1334	medium	O-H bending	alcohol
11	1505	strong	N-O stretching	Nitro compound
12	1438	medium	O-H bending	Carboxylic acid
13	1379	Strong	S=O stretching	Sulfonic acid
14	1274	strong	C=O stretching	alkyl aryl ether
15	1207	strong	C=O stretching	ester
16	1129	strong	C=O stretching	aliphatic ester
17	1037	strong	S=O stretching	sulfoxide
18	1013	Strong, broad	CO-O-CO stretching	anhydride
19	823	medium	C=C stretching	Alkene(tri-substituted)
